# A Novel Function of Sphingosine Kinase 2 in the Metabolism of Sphinga-4,14-Diene Lipids

**DOI:** 10.3390/metabo10060236

**Published:** 2020-06-08

**Authors:** Timothy Andrew Couttas, Yepy Hardi Rustam, Huitong Song, Yanfei Qi, Jonathan David Teo, Jinbiao Chen, Gavin Edmund Reid, Anthony Simon Don

**Affiliations:** 1 Centenary Institute, The University of Sydney, Camperdown, NSW 2006, Australia; t.couttas@centenary.org.au (T.A.C.); h.song@centenary.org.au (H.S.); j.qi@centenary.org.au (Y.Q.); j.teo@centenary.org.au (J.D.T.); j.chen@centenary.org.au (J.C.); 2 Department of Biochemistry and Molecular Biology, The University of Melbourne, Melbourne, VIC 3010, Australia; yrustam@student.unimelb.edu.au (Y.H.R.); gavin.reid@unimelb.edu.au (G.E.R.); 3 School of Chemistry, University of Melbourne, Parkville, VIC 3010, Australia; 4 Bio21 Molecular Science and Biotechnology Institute, The University of Melbourne, Melbourne, VIC 3010, Australia; 5 NHMRC Clinical Trials Centre, Faculty of Medicine and Health, The University of Sydney, Camperdown, NSW 2006, Australia

**Keywords:** sphingolipids, sphingadiene, sphingosine, mass spectrometry, sphingosine kinase, brain lipids, ceramide, sphingosine kinase 2, SphK2

## Abstract

The number, position, and configuration of double bonds in lipids affect membrane fluidity and the recruitment of signaling proteins. Studies on mammalian sphingolipids have focused on those with a saturated sphinganine or mono-unsaturated sphingosine long chain base. Using high-resolution liquid chromatography-tandem mass spectrometry (LC-MS/MS), we observed a marked accumulation of lipids containing a di-unsaturated sphingadiene base in the hippocampus of mice lacking the metabolic enzyme sphingosine kinase 2 (SphK2). The double bonds were localized to positions C4–C5 and C14–C15 of sphingadiene using ultraviolet photodissociation-tandem mass spectrometry (UVPD-MS/MS). Phosphorylation of sphingoid bases by sphingosine kinase 1 (SphK1) or SphK2 forms the penultimate step in the lysosomal catabolism of all sphingolipids. Both SphK1 and SphK2 phosphorylated sphinga-4,14-diene as efficiently as sphingosine, however deuterated tracer experiments in an oligodendrocyte cell line demonstrated that ceramides with a sphingosine base are more rapidly metabolized than those with a sphingadiene base. Since SphK2 is the dominant sphingosine kinase in brain, we propose that the accumulation of sphingadiene-based lipids in SphK2-deficient brains results from the slower catabolism of these lipids, combined with a bottleneck in the catabolic pathway created by the absence of SphK2. We have therefore uncovered a previously unappreciated role for SphK2 in lipid quality control.

## 1. Introduction

**Figure 1:**
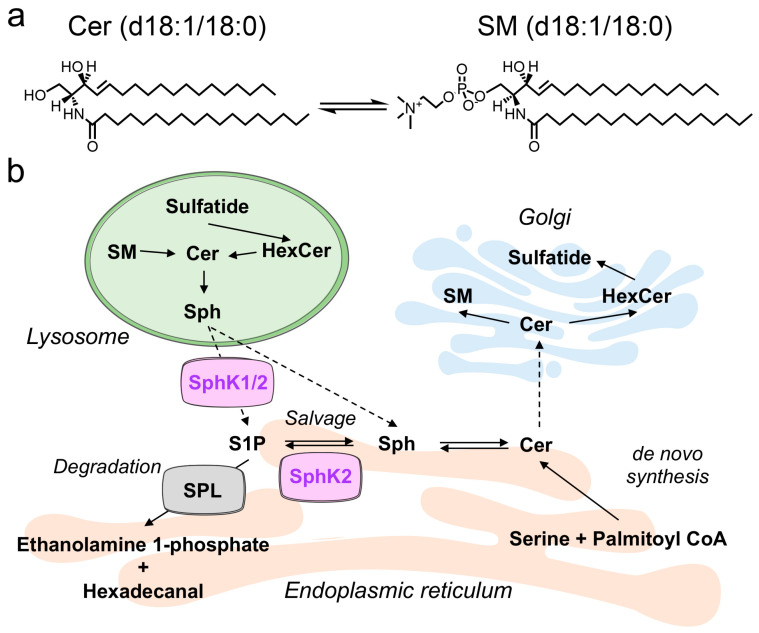
Sphingolipid structure and basic metabolism. (**a**) Structure of d18:1/18:0 ceramide (Cer) and sphingomyelin (SM). (**b**) Sphingolipid biosynthesis and breakdown. De novo synthesis of sphingolipids occurs in the endoplasmic reticulum, producing ceramides from serine and palmitoyl Coenzyme A (CoA). Ceramide transfer protein (CERT) transfers ceramides to the Golgi, where they are converted to SM and glycosphingolipids, such as hexosylceramide (HexCer) and sulfatide. Sphingolipid breakdown in lysosomes results in the generation of sphingosine (Sph), which can be recycled back to ceramide in the salvage pathway or phosphorylated by sphingosine kinase 1 (SphK1) or SphK2, forming sphingosine 1-phosphate (S1P). S1P can also be reutilized for sphingolipid synthesis in the salvage pathway or broken down in the endoplasmic reticulum by S1P lyase (SPL).

Sphingolipids are a large and diverse family of lipids, heavily enriched in the mammalian brain. They are important regulators of cell signaling and physiology in eukaryotes [1,2]. The common constituent of sphingolipids is their sphingoid base, which in mammals is predominantly 18-carbon sphingosine that contains a double bond at the C4–5 position (d18:1) (Figure 1a). Lipidomic studies often also quantify sphingolipids with the less abundant saturated dihydrosphingosine (d18:0) backbone [2].

The core diacyl sphingolipid structure is ceramide, which contains a sphingoid base amide-linked to a fatty-acyl chain (Figure 1a). These fatty-acyl chains vary from 14 to 26 carbons in length and are generally saturated or mono-unsaturated. The enzymatic addition of different headgroups to the primary hydroxyl of ceramide yields the different classes of sphingolipids, which include sphingomyelin (SM), hexosylceramide (HexCer) and sulfatide.

The length and degree of saturation of the sphingoid base and amide-linked acyl chains of sphingolipids can have a marked impact on their physiological function. For instance, d18:1/16:0 ceramide (C16 ceramide) antagonizes insulin signaling and promotes physiological insulin resistance, whereas its saturated counterpart C16 dihydroceramide (d18:0/16:0 ceramide), which lacks the C4–C5 double bond in the sphingoid base, does not [3]. In direct contrast to C16 ceramide, C24 ceramides (d18:1/24:0 and d18:1/24:1) protect against the development of insulin resistance in the liver [4,5]. The number, position, and configuration (cis or trans) of double bonds on both the sphingoid base and fatty-acyl chain greatly affects the membrane packing properties of sphingolipids, which can, in turn, affect their cellular signaling functions [6,7].

Tandem mass spectrometry with conventional collision-induced dissociation (CID) allows for the characterization of lipid headgroups, acyl chain length, and number of carbon–carbon double bonds, but provides no information on the position of the double bonds. Ultraviolet photodissociation-tandem mass spectrometry (UVPD-MS/MS) is an emerging approach for structural characterization of lipids in which high-energy excitation through absorption of 193 nm UV photons causes direct dissociation, yielding product ion and structural data that are not observed with conventional CID [8,9,10,11,12]. Importantly, 193 nm UV excitation results in characteristic dissociation of bonds adjacent to carbon–carbon double bonds in lipid acyl chains, allowing the location of double bonds within the acyl chains to be determined.

Phosphorylation of sphingosine and other sphingoid bases by sphingosine kinases 1 and 2 (SphK1 and 2) forms the lipid signaling molecule sphingosine 1-phosphate (S1P) and related sphingoid base phosphates [13,14]. S1P executes a wide array of cell signaling roles both through a family of five G-protein coupled receptors that are specific to S1P [13,15] and through specific intracellular S1P binding targets, such as histone deacetylases [16]. In addition to forming the signaling molecule S1P, sphingosine phosphorylation is an essential and evolutionarily-conserved step in the catabolism of all sphingolipids. This proceeds through irreversible degradation of S1P by S1P lyase, forming hexadecenal and ethanolamine phosphate (Figure 1b) [1,2].

As the only pathway for complete catabolism of sphingolipids, sphingosine kinases and S1P lyase are key regulators of sphingolipid homeostasis [17]. In mice, loss of S1P lyase is lethal within a few weeks of weaning, attributed to a range of gross developmental defects [18,19] and pronounced deregulation of lipid levels in multiple organs [20]. The absence of S1P lyase causes increased sphingolipid biosynthesis via the salvage pathway, which is associated with reduced de novo sphingolipid biosynthesis, thus causing widespread disruption to lipid metabolism [21]. Loss of both SphK1 and SphK2 is also lethal; however, the mice die with defects in vasculogenesis and neurogenesis attributed to the loss of S1P signaling functions [22]. Mice lacking either SphK1 or SphK2 exhibit no obvious phenotypic abnormalities.

We have previously reported the loss of S1P and SphK2 activity early in the pathogenesis of Alzheimer’s disease [23], and recently demonstrated that loss of SphK2 sensitizes to hippocampal atrophy and myelin loss in a mouse model of Alzheimer’s disease [24]. We have also shown heightened anxiety in mice lacking SphK2 [25]. SphK2 catalyzes the majority of S1P synthesis in the brain [25], and an important role for SphK2 in protection against brain atrophy following cerebral ischemia has been established [26]. Here, we report pronounced and selective accumulation of sphingolipids containing a second double bond in the sphingoid base, termed sphingadienes, in the hippocampus of SphK2 knockout (SphK2^−/−^) mice. Employing the emerging technique of UVPD-MS/MS, the double bonds were unambiguously assigned to positions C4–C5 and C14–C15 in the sphingoid base backbone of these lipids. In metabolic tracing experiments, we show that these sphingadiene-containing lipids are catabolized more slowly than the more abundant sphingosine-based sphingolipids, and, therefore, propose that sphingadiene lipids accumulate in the brains of SphK2^−/−^ mice due to poor degradation via the sphingosine kinase-S1P lyase pathway.

## 2. Results

### 2.1. Accumulation of Sphingadiene Lipids in the Hippocampus of SphK2 Deficient Mice

**Figure 2:**
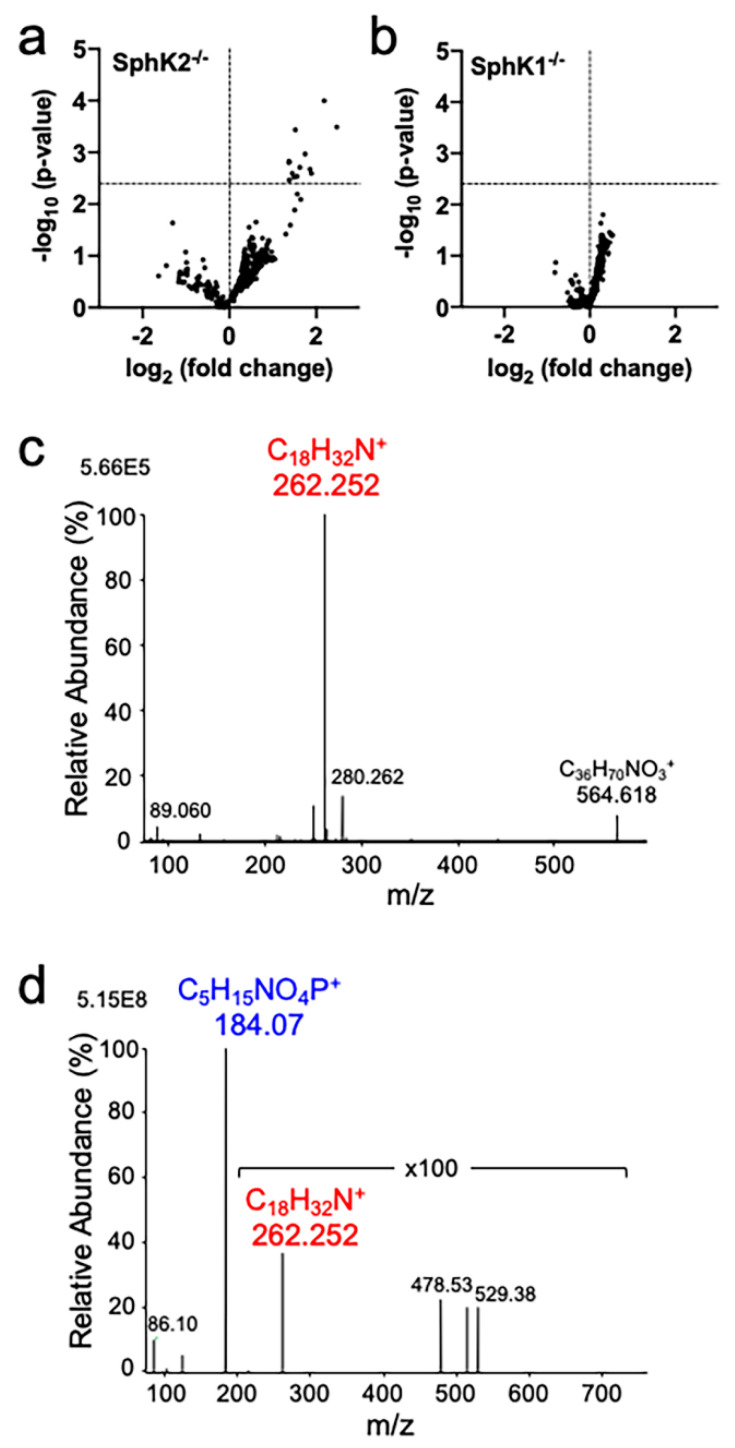
Sphingadiene-containing lipids are increased in hippocampus of SphK2^−/−^ mice. (**a**,**b**) Volcano plots showing *p*-value against fold change for each lipid identified in (**a**) SphK2^−/−^ and (**b**) SphK1^−/−^ mice, relative to wild-type (WT) control littermates (mean fold-change from six mice per group). The dotted line shows the threshold for statistical significance at Q < 0.1. Identities of sphingolipids significantly upregulated in SphK2^−/−^ mice are given in Table 1. (**c**,**d**) Q-Exactive HF-X CID-MS/MS product ion spectra for [M + H]^+^ ions of (**c**) d18:2/18:0 ceramide and (**d**) d18:2/18:0 SM. Assignment as d18:2/18:0 ceramide was based on precursor ion *m*/*z* 564.534 and product ion *m*/*z* 262.252, which is diagnostic for sphingadiene. The *m*/*z* 184.073 choline phosphate product ion together with precursor ion *m*/*z* 729.590 is diagnostic of d36:2 SM, and the presence of the *m*/*z* 262.252 product ion allowed for the assignment of this lipid as d18:2/18:0 SM.

Untargeted lipidomic (LC-MS/MS) analysis of hippocampus tissue from SphK1^−/−^ and SphK2^−/−^ mice, along with wild-type (WT) control littermates for each, identified phospholipids (phosphatidylcholine, PC; phosphatidylethanolamine, PE; phosphatidylserine, PS), sphingolipids (ceramide, Cer; sulfatide, ST; hexosylceramide, HexCer; sphingomyelin, SM), and neutral (diacylglycerol, DAG; and triacylglycerol, TAG) lipid species. Lipids identified using LipidSearch software were manually verified for peak shape and correct product ions, resulting in a total of 357 confirmed lipids in SphK2^−/−^ and 363 in SphK1^−/−^ mice. After adjusting for the false discovery rate, a total of thirteen lipids were significantly increased in SphK2^−/−^ compared to WT mice (Figure 2a and Table 1). In contrast, no lipids differed significantly in abundance between SphK1^−/−^ mice and their WT littermates (Figure 2b). All thirteen lipids whose levels were significantly increased in SphK2^−/−^ mice were sphingolipids containing a sphingadiene (18:2) long chain base, as identified by the diagnostic *m*/*z* 262.252 product ion (expected *m*/z 262.253) that distinguishes sphingadiene from sphingosine (*m*/*z* 264.269) and dihydrosphingosine (*m*/*z* 266.284). CID-MS/MS product ion data for 18:2/18:0 ceramide and SM are illustrated as examples (Figure 2c,d). The C18:0 N-acyl chain was the most abundant fatty acyl chain in brain ceramides and SM. The full dataset is available as Supplementary Tables S1 and S2.

**Figure 3:**
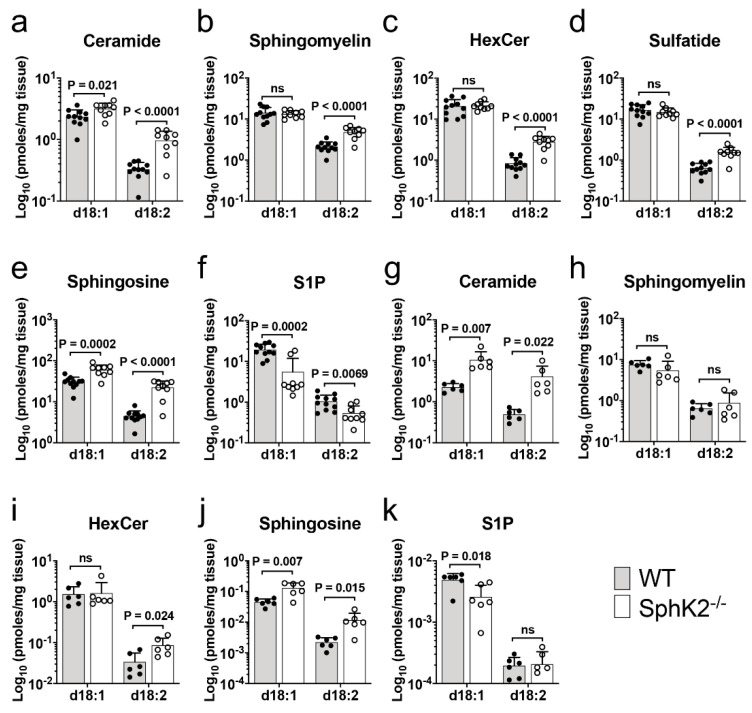
Increased sphingadienes in the hippocampus and liver of SphK2^−/−^ mice. (**a**–**f**) Total levels of d18:1 and d18:2 ceramide (**a**), SM (**b**), HexCer (**c**), sulfatide (**d**), sphingosine (**e**), and S1P (**f**), in hippocampus extracts of WT (shaded bar) and SphK2^−/−^ (clear bar) mice at 6 months of age (n = 11, WT; n = 9, SphK2^−/−^ mice per group), determined by targeted triple quadrupole LC-MS/MS analysis. (**g**–**k**) Levels of d18:1 and d18:2 ceramide (**g**), SM (**h**), HexCer (**i**), sphingosine (**j**), and S1P (**k**), in livers of WT (shaded bar) and SphK2^−/−^ (clear bar) mice at 8 months of age (n = 6 mice per group). Statistical significance was determined by unpaired *t*-tests.

Our untargeted lipidomic data was validated by targeted LC-MS/MS on hippocampus samples from an independent cohort of 6-month-old SphK2^−/−^ mice. Total levels of ceramide, SM, HexCer, and sulfatide containing d18:2 sphingadiene backbones were 3.0-, 2.2-, 3.4-, and 2.4-fold higher (all *p* < 0.0001), respectively, in SphK2^−/−^ compared to WT mice (Figure 3a–d). The increase in sphingadiene lipid levels in SphK2^−/−^ mice was observed for all individual acyl chain variants (Supplementary Table S3). In contrast, the total level of d18:1 ceramides increased by a more modest 1.3-fold (*p* = 0.021) in SphK2^−/−^ hippocampus samples, and there was no significant difference in total levels of d18:1 SM, HexCer, and sulfatide (Figure 3a–d). Sphingosine and sphingadiene were 2- and 5-fold higher, respectively, in the hippocampus of SphK2^−/−^ mice (Figure 3e, both *p* < 0.001); while S1P and sphingadiene 1-phosphate were 3.5- (*p* = 0.0002) and 2-fold (*p* = 0.007) lower, respectively (Figure 3f). The 72% reduction in d18:1 S1P in SphK2^−/−^ mouse brains is in agreement with prior data showing that SphK2 is responsible for the majority of S1P synthesis in the brain [25].

To determine if SphK2^−/−^ mice accumulate sphingadiene lipids in another tissue that expresses relatively high levels of SphK2 [27], we also quantified sphingolipids in liver. Ceramides with a d18:1 sphingoid base were 5-fold higher (*p* = 0.007), while those with a d18:2 base were 8-fold higher (*p* = 0.022) in SphK2^−/−^ compared to WT mouse livers (Figure 3g). SM and HexCer with d18:1 sphingoid bases, as well as d18:2 SM species, did not differ significantly between the two genotypes, whereas d18:2 HexCer was 2.5-fold higher (*p* = 0.024) in the SphK2^−/−^ livers (Figure 3h,i and Supplementary Table S4). Sphingosine and sphingadiene levels were 3-fold (*p* = 0.007) and 5-fold (*p* = 0.015) higher (Figure 3j), while S1P was 2-fold lower (*p* = 0.018) in SphK2^−/−^ liver tissue (Figure 3k). Sphingadiene 1-phosphate was not significantly altered in SphK2^−/−^ liver tissue. Therefore, a preferential increase in d18:2 relative to d18:1 sphingolipids was observed in livers of SphK2-deficient mice; however, the difference was more pronounced in brain.

### 2.2. UVPD-MS/MS Assigns Double Bonds to the C4 and C14 Position of Sphingadiene

**Figure 4:**
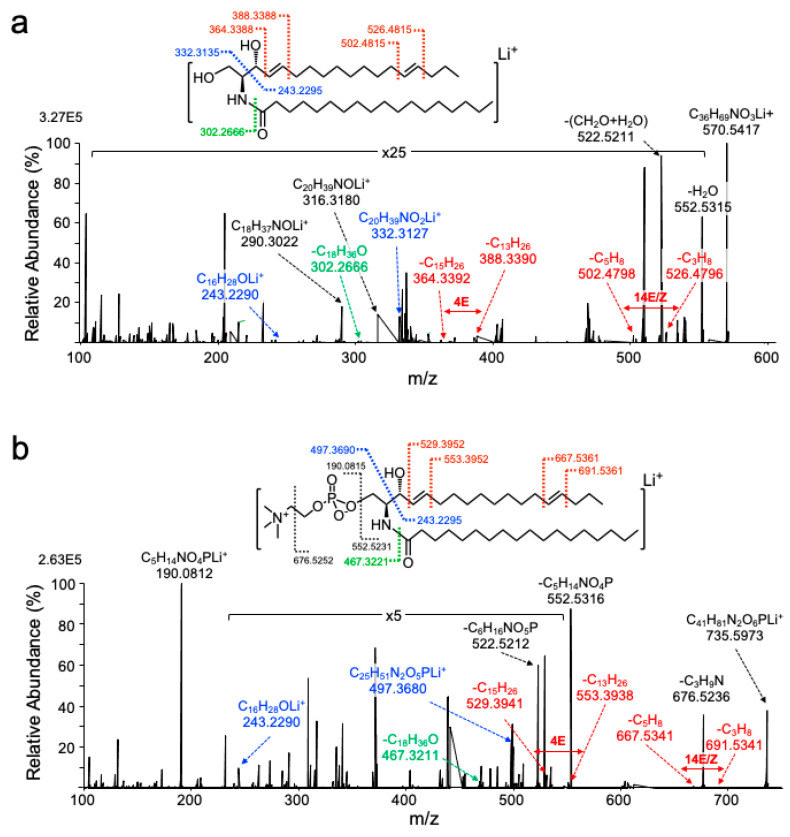
One hundred and ninety-three-nanometer ultraviolet photodissociation-tandem mass spectrometry (UVPD-MS/MS) spectra for 18:2 ceramide and SM. Product ion spectra resulting from UVPD-MS/MS of [M + Li]^+^ precursor ions of (**a**) d18:2(4,14)/18:0 ceramide and (**b**) d18:2(4,14)/18:0 SM. The chemical structure above each spectrum shows points of cleavage, labeled with the expected *m*/*z* for the resulting product ions. Structurally diagnostic product ions for sphingoid backbone (blue), N–C amide bond (green) and specific product ions resulting from cleavage of the sphingoid base double bonds (red) have been highlighted.

To pinpoint the position of the double bonds in the major brain sphingadiene lipids, we employed 193 nm UVPD-MS/MS on a custom modified high-resolution Orbitrap instrument. Precursor ions at *m*/*z* 570.542 and 735.597 corresponded to the expected [M + Li]^+^ ions for d18:2/18:0 ceramide and SM, respectively (Figure 4). UVPD-MS/MS of the *m*/*z* 570.542 precursor ion (Figure 4a) yielded product ions at *m*/*z* 243.229 and 332.313, as expected for UV-induced cleavage between C2 and C3 of the sphingadiene base. A pair of product ions differing by 24.000 mass units (*m*/*z* 364.339 and 388.339) were diagnostic for the C4–C5 double bond that is typical of sphingosine. Another pair differing by 24.000 mass units (*m*/*z* 502.480 and 526.480), corresponding to the loss of C_5_H_8_ and C_3_H_8_ resulting from the cleavage of the C13–14 and C15–C16 bonds in the long chain base (Figure 4a), confirm the presence of the second unsaturated bond in the sphingoid base chain and localize it to the C14–C15 position. The product ion at *m*/*z* 302.2666, resulting from the cleavage of the amide bond and subsequent loss of C_18_H_36_O, confirms the C18:0 saturated acyl-chain amide-linked to sphingadiene. All product ion masses matched the expected masses to within 4 parts per million (ppm).

UVPD-MS/MS of the *m*/*z* 735.597 [M + Li]^+^ d18:2/18:0 SM ion yielded products at *m*/*z* 243.229 and 497.369, resulting from the cleavage between C2 and C3 of the sphingoid base (Figure 4b). Two pairs of product ions differing by 24.000 mass units were also observed from cleavage at the C4–C5 (*m*/*z* 529.394 and 553.394) and C14–C15 positions (*m*/*z* 667.534 and 691.534), indicative of a sphinga-4,14-diene backbone. The C18:0 N-acyl chain was confirmed by the amide bond fragmentation product ion with *m*/*z* 467.322, which corresponds to a loss of C_18_H_36_O. A strong signal was detected for product ion *m*/*z* 190.081, which corresponds to the lithium adduct of the SM choline–phosphate headgroup.

### 2.3. SphK1 and SphK2 Exhibit Similar Affinity for Sphingosine and Sphinga-4,14-Diene

**Figure 5:**
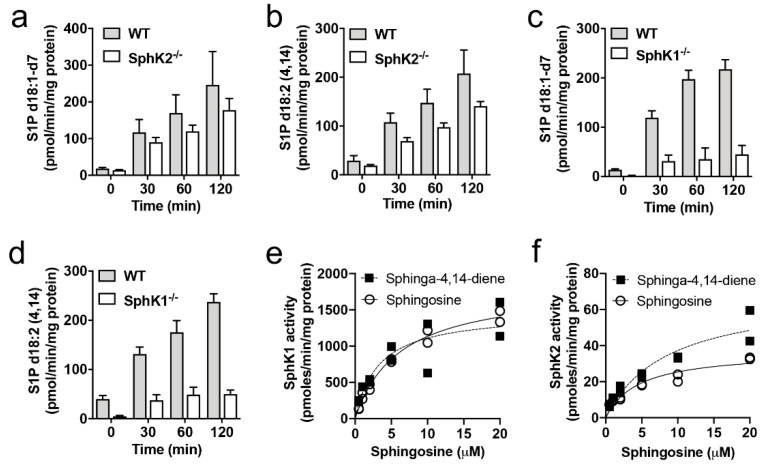
SphK1 and SphK2 show a similar affinity for sphingosine and sphingadiene. (**a**–**d**) Sphingosine kinase activity of SphK2^−/−^ (**a**,**b**) or SphK1^−/−^ (**c**,**d**) mouse brain lysates and WT littermate controls, with D7-sphingosine (**a**,**c**) or sphinga-4,14-diene (**b**,**d**) as substrates. Results show mean and standard error for triplicate assays. (**e**) SphK1 and (**f**) SphK2 activity as a function of sphingosine (d18:1; open circles) or sphingadiene (d18:2; closed squares) concentration, measured using recombinant human SphK1 or SphK2. Michaelis–Menten constants (K_m_) and maximal reaction rates (V_max_) were calculated for each substrate using non-linear regression, and are provided in the text.

SphK2 is quantitatively more important for S1P synthesis in the brain than SphK1 (Figure 3f) [25] and demonstrates broader substrate specificity, as it is able to catalyze the phosphorylation of phytosphingosine and the sphingoid base analog Fingolimod, as well as sphingosine and dihydrosphingosine [27,28]. As sphingosine kinases catalyze an essential step in the catabolism of all sphingolipids, we assessed whether the accumulation of sphingadiene-containing lipids might be attributed to the poor affinity of SphK1 for sphingadiene as a substrate. This would result in selective accumulation of sphingadiene lipids in the absence of SphK2. We, therefore, compared phosphorylation of sphingosine and sphinga-4,14-diene over time in brain lysates of SphK1^−/−^ or SphK2^−/−^ mice. Sphingosine kinase activity in these mice is wholly dependent on SphK2 or SphK1, respectively. Deuterated sphingosine (D7-sphingosine) was used to avoid interference from endogenous sphingosine and S1P present in the brain lysates used as the source of enzyme for the reactions. No deuterated standard was available for sphingadiene, and we, therefore, used the unlabeled standard. Both SphK1 (SphK2^−/−^ brain extracts) and SphK2 (i.e., SphK1^−/−^ brain extracts) phosphorylated sphingadiene with similar efficiency to sphingosine (Figure 5a–d). The in vitro phosphorylation rate was much lower in SphK1^−/−^ than SphK2^−/−^ brain extracts. This is in agreement with a recent publication reporting that SphK1 is much more efficient than SphK2 in the in vitro sphingosine kinase activity assay, whereas SphK2 deficiency has a much greater effect than SphK1 deficiency on neuronal S1P levels [29].

We also investigated the affinity and maximal reaction rate of recombinant human SphK1 and SphK2 for sphingosine and sphinga-4,14-diene. The Michaelis constant (K_m_) and maximum reaction rate (V_max_) for sphingosine were 6.2 μM and 1832 pmol/min/µg for SphK1, and 4.6 μM and 36.98 pmol/min/µg for SphK2 (Figure 5e,f). These K_m_ values correspond well with previously reported values for SphK1 (5–17 μM) and SphK2 (3–5 μM) [27,30,31,32]. The K_m_ and V_max_ values for sphinga-4,14-diene were 3.2 μM and 1469 pmol/min/µg for SphK1, and 8.5 μM and 69.10 pmol/min/µg for SphK2 (Figure 5e,f). Therefore, the accumulation of sphingadiene-containing lipids in the absence of SphK2 cannot be explained by the poor affinity of SphK1 for sphingadiene.

### 2.4. Sphingadiene (d18:2) Ceramides Are Metabolized More Slowly Than d18:1 Ceramides

**Figure 6:**
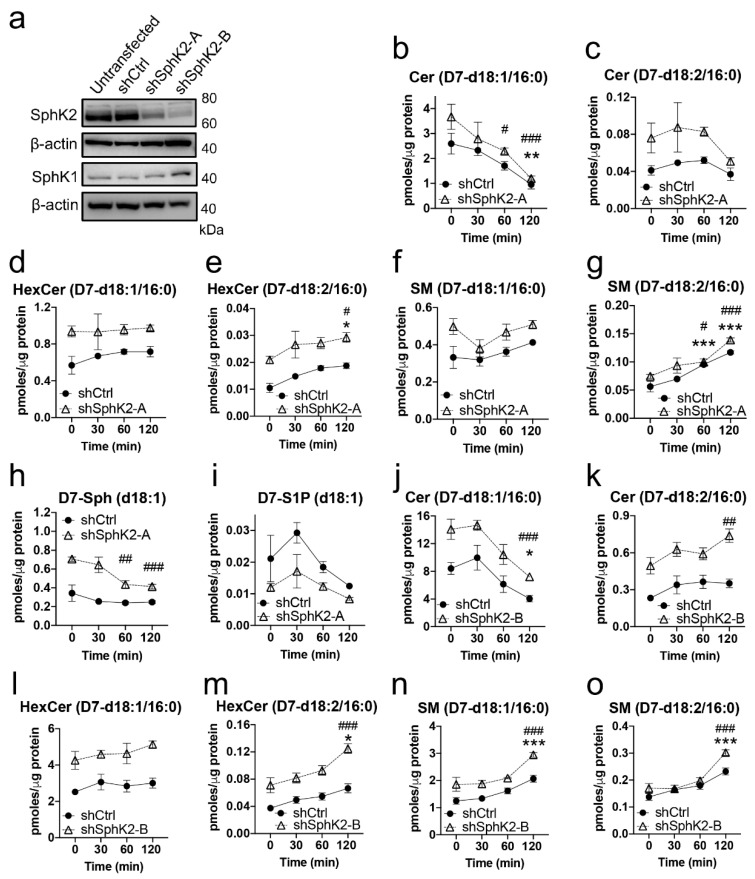
d18:1 ceramides are metabolized more rapidly than d18:2 ceramides. (**a**) Western blots for SphK2 and SphK1 protein levels in untransfected, shCtrl, shSphK2-A, and shSphK2-B MO3.13 cell pools. (**b**–**g**) Levels of D7-d18:1/16:0 (**b**,**d**,**f**) and D7-d18:2/16:0 (**c**,**e**,**g**) ceramide (Cer) (**b**,**c**), HexCer (**d**,**e**), and SM (**f**,**g**) over time, following 2 h loading of shCtrl or shSphK2-A cells with D7-sphingosine (1 µM). (**h**,**i**) Levels of D7-sphingosine and D7-S1P in shCtrl or shSphK2-A cells over time. (**j**–**o**) Levels of D7-d18:1/16:0 (**j**,**l**,**n**) and D7-d18:2/16:0 (**k**,**m**,**o**) Cer (**j**,**k**), HexCer (**l**,**m**), and SM (**n**,**o**) over time, following 2 h loading of shCtrl or shSphK2-B cells with D7-sphingosine (1 µM). Closed circles (•): shCtrl; open triangles (△): shSphK2. Statistical significance was determined by 2-way ANOVA, and Dunnett’s post-test was applied to compare lipid levels at 30, 60, or 120 min to the 0 min time point. * *p* < 0.05; ** *p* < 0.01, *** *p* < 0.001 relative to the 0 time point in shCtrl cells; # *p* < 0.05; ## *p* < 0.01, ### *p* < 0.001 relative to the 0 time point in shSphK2 cells. Overall ANOVA results for shCtrl vs. shSphK2 cells are reported in Results.

We next tested if the accumulation of sphingadiene-containing lipids in the absence of SphK2 could be attributed to a slower turnover of these lipids in comparison to their more abundant d18:1 counterparts. This could cause selective accumulation of sphingadiene-containing lipids in SphK2^−/−^ brains, as the rate of catabolism for all sphingolipids is predicted to be slower in the absence of SphK2. As the majority of brain lipids are synthesized by oligodendrocytes [33,34], we performed a pulse–chase experiment with the oligodendrocyte cell line MO13.3. To test the role of SphK2 in the metabolism of labeled sphingolipids, we generated cells stably expressing shRNA against SphK2 (shSphK2) and non-silencing shRNA control cells (shCtrl). Effective silencing of SphK2 with two distinct shRNA sequences (shSphK2-A and shSphK2-B) was confirmed by Western blot (Figure 6a). Some compensatory upregulation of SphK1 was apparent in shSphK2-B cells, presumably as these cells showed the lowest SphK2 expression. However, we noted that SphK1 protein was expressed at low levels in these cells, requiring long exposure time for the membrane in comparison to other cell lines.

Cells were loaded with deuterated (D7) sphingosine for 2 h, and the levels of D7-labeled sphingolipids were quantified at 0, 30, 60, and 120 min after removing the tracer from the culture medium. D7-labeled ceramide, HexCer, and SM with both sphingosine and sphingadiene backbones were observed. Over 70% of these contained a C16:0 N-acyl chain, and we have, therefore, shown levels of these C16:0 lipids over time (Figure 6b–o). Levels of D7-d18:1/16:0 ceramide, SM, and HexCer were much higher than their D7-d18:2 equivalents in both shCtrl and shSphK2-A cells (Figure 6b–g), in agreement with the equilibrium between d18:1 and d18:2 sphingolipids strongly favoring the former. Both D7-d18:1 and D7-d18:2 sphingolipids were higher in shSphK2-A cells compared to shCtrl cells (*p* < 0.001 by 2-way ANOVA for d18:1/16:0 ceramide, d18:2/16:0 ceramide, d18:1/16:0 HexCer, d18:2/16:0 HexCer, and d18:1/16:0 SM; *p* = 0.0025 for d18:2/16:0 SM), while D7-S1P was lower in shSphK2-A compared to shCtrl cells (Figure 6i; *p* < 0.0001 by 2-way ANOVA). The higher levels of D7-ceramide, D7-SM, and D7-HexCer in shSphK2 cells are likely explained by a lack of deuterated lipid flux through SphK2. Levels of D7-sphingosine remained relatively constant in the shCtrl cells but declined significantly at 60 and 120 min in shSphK2-A cells (Figure 6h). D7-sphingadiene was not detected, suggesting that the ∆14 desaturase that produces sphinga-4,14-diene acts on ceramide, rather than sphingosine, as its substrate.

Over the course of 2 h after removal of the D7-sphingosine tracer, D7-d18:1/16:0 ceramide declined 2.7-fold in shCtrl (*p* = 0.008), and 3.1-fold in shSphK2-A cells (*p* = 0.0001) (Figure 6b). In contrast, levels of D7-d18:2/16:0 ceramide did not decline significantly over the 2 h time course (Figure 6c). Levels of D7-d18:1/16:0 HexCer and D7-d18:1/16:0 SM were not significantly altered (Figure 6d,f), whereas both D7-d18:2/16:0 HexCer and D7-d18:2/16:0 SM increased significantly over the 2 h time course in both shCtrl and shSphK2-A cells (Figure 6e,g).

Similar results were observed upon repeating the tracer experiment with MO3.13 cells expressing a different shRNA sequence, shSphK2-B (Figure 6j–o). Levels of both D7-d18:1/16:0 and D7-d18:2/16:0 sphingolipids were higher in shSphK2-B than shCtrl cells. D7-d18:1/16:0 ceramide declined in both shCtrl and shSphK2-B cells (*p* = 0.032 in the WT cells, *p* = 0.0008 in shSphK2), whereas D7-d18:2/16:0 ceramide increased 1.5-fold in both shSphK2-B cells and shCtrl cells (*p* = 0.009 in shSphK2-B; not significant, *p* = 0.28, in shCtrl) over the 2 h time course following the removal of the D7-sphingosine (Figure 6k). D7-d18:1/16:0 HexCer levels did not change significantly over the 2 h time course, whereas D7-d18:2/16:0 HexCer increased significantly in both cell lines (Figure 6l–m). In this experiment, both D7-d18:1/16:0 and D7-d18:2/16:0 SM increased significantly over the 2h time course (Figure 6n–o). Overall, these results demonstrate that d18:1 ceramides are more rapidly metabolized than d18:2 ceramides, which we propose is attributed to faster catabolism of d18:1 ceramides.

SphK2 silencing did not affect the viability of MO3.13 cells cultured in serum-containing medium (90.0 ± 4.9% viability for shCtrl, 96.5 ± 6.1% for shSphK2-A and 94.0 ± 0.87% for shSphK2-B; mean ± SD; n = 3 three cell counts) or serum-free oligodendrocyte differentiation medium (98.8 ± 2.1% viability for shCtrl, 96.8 ± 5.5% for shSphK2-A and 98.8 ± 2.1% for shSphK2-B; mean ± SD; n = 3 three cell counts).

## 3. Discussion

Sphingosine kinases fulfill dual biochemical functions in mammalian biology. First, they produce the physiologically-essential signaling molecule S1P, which signals both through a family of five G-protein coupled receptors and through intracellular binding targets. Second, they catalyze the penultimate step in the catabolism of sphingolipids, as phosphorylation of sphingoid bases is necessary for their degradation by S1P lyase [1,13,15]. Here, we established that sphingolipids with a sphingadiene long chain base accumulate in the hippocampus of SphK2^−/−^ but not SphK1^−/−^ mice. Using the emerging fragmentation method of UVPD-MS/MS, we localized the double bonds to the C4–C5 and C14–C15 positions of the sphingoid base chain, allowing us to test the rate of phosphorylation of this sphingoid base by SphK1 and SphK2 in vitro. There was no difference in the capacity for either SphK1 or SphK2 to phosphorylate sphinga-4,14-diene compared to sphingosine. Instead, we showed that D7-labelled d18:1 ceramide levels decline over a 2 h time course in MO3.13 cells, whereas d18:2 ceramide levels do not. Whereas d18:1 HexCer levels were relatively unchanged over the 2 h time course, d18:2 HexCer levels increased. As the equilibrium between d18:1 and d18:2 sphingolipids favors d18:1, these differences in the rates of metabolism imply that ceramides with a sphinga-4,14-diene backbone are less efficiently catabolized than the more common sphingosine-based lipids.

Our findings are strongly supported by a very recent publication showing that, compared to S1P, sphinga-4,14-diene 1-phosphate is less efficiently catabolized by S1P lyase in vitro (using cell extracts) [35]. Our data extend this work by demonstrating that sphingadiene-containing lipids are more slowly catabolized than sphingosine-containing lipids in living cells. Similarly, S1P lyase mutants in the fly *Drosophila melanogaster* accumulate sphinga-4,6-diene, which is speculated to contribute to muscle degeneration [36]. In agreement with our data, Jojima et al. [35] also found that both SphK1 and SphK2 catalyze sphingadiene phosphorylation as efficiently as sphingosine in vitro. Since SphK2 catalyzes the bulk of S1P synthesis in the brain [24,25,29], its absence shifts the equilibrium between sphingoid bases (sphingosine, sphingadiene) and their respective 1-phosphates in favor of the sphingoid bases. This is expected to result in more efficient re-acylation of these sphingoid bases to ceramides in the salvage pathway (Figure 1), producing the increase in ceramide levels observed in both brain and liver (Figure 3). Our findings, both in vitro and in vivo, indicate that this occurs to a greater extent with sphingadiene relative to sphingosine. We propose two possible explanations for this, although noting that these are speculative at this stage: (i) Since S1P lyase favors S1P over sphingadiene 1-phosphate as a substrate [35], more sphingadiene 1-phosphate is available for dephosphorylation to sphingadiene by S1P phosphatases 1 and 2 (Sgpp1 and 2) and recycling into ceramides [37,38,39]. (ii) The sub-cellular localization of sphingosine-containing and sphingadiene-containing lipids differs in such a way that sphingosine-containing lipids are more readily degraded through the SphK2-S1P lyase pathway.

Techniques for assignment of double bond position in lipids without chemical derivatization have only recently been developed and are not yet used in routine LC-MS/MS analyses. UVPD-MS/MS has been used for isomer differentiation and localization of double bonds in glycerophospholipids [8], lipooligosaccharides [9,11], gangliosides [10], and sphingolipids [12]. However, this is the first report employing UVPD-MS/MS in combination with genetic models to answer specific biological questions concerning lipid metabolism and function. UVPD-MS/MS does not require any derivatization, modification, or chromatographic separation to resolve sites of unsaturation, making this a powerful technique that can be readily incorporated into lipidomic workflows. An alternative MS/MS approach for double bond assignment is ozonolysis, which coverts alkene bonds to ozonoids that fragment into product ion pairs diagnostic of double bond position [40,41]. However, an ozone source may create unwanted hazards. The ionization efficiency of ozonolysis is reportedly reduced in positive ion mode, and the high capillary voltage reduces sensitivity [42]. Comprehensive structural characterization of lipids can also be performed using electron-induced dissociation (EID) in combination with differential ion mobility spectrometry (also referred to as electron-impact excitation of ions from organics (EIEIO)) [43,44]. Fragment spectra from EID can identify lipid class, number of double bonds, and regio-isomers, although the precise position(s) of double bonds can be difficult to isolate using EID, since single bond fragments interfere with double bond diagnostic peaks [43]. An important caveat regarding the general applicability of UVPD-MS/MS and other complex dissociation techniques described above is the complexity of data analysis. Even with CID, lipidomic analysis is very complex and requires both training and significant knowledge of lipid structures. Analyzing fragments related to double bond positions increases this complexity and may be challenging to implement into broad lipidomic profiling experiments.

Our assignment of the second double bond in murine brain sphingadiene to the C14–C15 position is in agreement with three publications from the late 1960s in which chemical derivatization techniques coupled to thin-layer or gas chromatography were used to determine double bond positions in d18:2 SM species of human plasma and aorta samples [45,46,47]. Despite this, the levels and functional significance of sphingadiene-containing lipids have been very sparsely investigated relative to the sphingoid bases sphingosine and sphinganine (dihydrosphingosine), and they are often not included in routine lipidomic analyses. It was very recently shown that d18:2 is the second most abundant sphingoid base in human plasma [48] and that levels of sphinga-4,14-diene in mice are highest in kidney, followed by brain, lung, and then colon [35]. Sphingadienes with double bonds in the C4–C5 and C8–C9 positions of the sphingoid base chain are common in plants and fungi, such as soybean, corn, wheat, and yeast [49,50]. Naturally occurring sphingadienes inhibit both chemically- and genetically-induced colon cancer development in mice [50,51,52], associated with disrupted Akt membrane translocation and signaling [52], and reduced Wnt transcriptional activity [51]. Sphingadienes have also been shown to suppress the growth of neuroblastoma xenograft tumors [53].

The introduction of the C4–C5 trans double bond into ceramide is catalyzed by Δ4-dihydroceramide desaturase (DEGS1) [54]. Two research teams very recently identified FADS3 as the desaturase catalyzing the addition of the C14–C15 double bond in the sphingosine long chain base [35,48]. Jojima et al. [35] presented evidence indicating that the second double bond is introduced into ceramide, not sphingosine. Our data also suggested that desaturation at the C14–C15 bond occurs in ceramide, not sphingosine, as D7-sphingadiene was below the limit of detection in cell culture, despite abundant levels of D7-sphingosine and clearly detectible D7-d18:2 sphingolipids. In contrast, Karsai et al. [48] showed that FADS3 can directly desaturate sphingosine in vitro, however, inhibiting ceramide synthesis with Fumonisin B1 greatly reduced sphingadiene formation in living cells. Mice lacking FADS3 have reduced brain docosahexaenoic acid levels but were reported to display no major phenotypic abnormalities [55]. However the International Mouse Phenotyping Consortium (https://www.mousephenotype.org) [56] reported impaired auditory response, decreased circulating phosphates, and increased serum albumin in FADS3 knockout mice.

The C14–C15 double bond in sphinga-4,14-diene is a cis bond [14], which introduces a kink in the acyl chain and is thereby expected to decrease the packing density of the lipid bilayer. Accordingly, Jojima et al. [35] showed that a lower proportion of d18:2 HexCer localizes to detergent-resistant membrane domains compared to d18:1 HexCer. The brain is enriched with sphingolipids, so an increased proportion of d18:2(4,14) sphingolipids may have important consequences for neuronal lipid domains and myelin integrity. We have reported that SphK2 deficiency synergizes with an amyloid-producing transgene to create severe myelin deficits [24]. It is possible that myelin structure and function is destabilized by an increased abundance of sphingadiene lipids in SphK2^−/−^ mice, and this should be investigated in future studies, potentially by crossing SphK2^−/−^ to FADS3^−/−^ mice. Future studies will also investigate whether an increased sphingadiene lipid burden sensitizes oligodendrocytes to cell death.

In conclusion, we have identified significant accumulation of sphingolipids containing a sphingadiene base in the hippocampus of mice lacking functional SphK2 and have employed the emerging technique of UVPD-MS/MS to assign double bond positions to the C4–C5 and C14–C15 positions. To the best of our knowledge, this is the first study to apply UVPD-MS/MS towards an improved understanding of metabolic pathways and enzyme function. The biology of sphingadiene-containing lipids has not been elucidated, and these lipids have not commonly been assayed in targeted lipidomic analyses. Sphingosine kinases remain targets of interest for cancer therapy, and inhibitors of both SphK1 and SphK2 have been investigated in clinical trials. Our findings demonstrate the importance of analyzing and better understanding the functional significance of sphingadiene lipids, particularly given that circulating levels of these lipids were reported as significantly higher in females [48]; and knockout of SphK2, or Bax and Bak [57], has now been shown to alter the balance between sphingadiene- and sphingosine-containing lipids.

## 4. Materials and Methods

### 4.1. Lipid Standards

All lipids, with the exception of 17:0/17:0/17:0 triacylglycerol (TAG) (Cayman Chemical, Ann Arbor, MI, USA), were purchased from Avanti Polar Lipids (distributed by Sigma Aldrich, Castle Hill, NSW, Australia). All lipid extractions were spiked with an internal standard mixture comprising of 0.2 nmole of d17:1 Sph (860640) and S1P (860641), and 1 nmole of each of the following: d18:1/12:0 SM (860583), d18:1/17:0 ceramide (860517), 18:1/12:0 glucosylceramide (860543), d18:1/12:0 sulfatide (860573), 19:0/19:0 phosphatidylcholine (850367), 17:0/17:0 phosphatidylethanolamine (830756), 17:0/17:0 phosphatidylserine (840028), 17:0/17:0 d5-diacylglycerol (110580), 17:0/17:0 phosphatidylglycerol (830456), and 17:0/17:0/17:0 TAG (16489). Deuterated (D7) sphingosine (860657), d18:1 sphingosine (860490), and d18:2 sphinga-4,14-diene (860665) were purchased from Avanti Polar Lipids.

### 4.2. Mouse Tissue Samples

Mice lacking functional SphK1 (SphK1^−/−^) or SphK2 (SphK2^−/−^) were derived by breeding heterozygous parents (i.e., SphK1^+/−^ × SphK1^+/-^ or SphK2^+/−^ × SphK2^+/−^), allowing us to compare SphK1^−/−^ and SphK2^−/−^ mice to their WT littermates (i.e., SphK1^+/+^ or SphK2^+/+^). Genotyping was performed as described previously [22]. Mice were housed in ventilated cages with enrichment consisting of nesting material and a plastic dome, and were given access to food and water ad libitum. For untargeted mass spectrometry analysis, we used groups of 6 male SphK2^−/−^ and WT mice at 8 months of age, or 6 (3 male and 3 female) SphK1^−/−^ and 8 (3 male and 5 female) WT mice at 6 months of age. A separate cohort of male SphK2^−/−^ (n = 9) and WT (n = 11) mice, aged 6 months, were used for targeted lipidomic analysis of the hippocampus. The research was approved by the Animal Care and Ethics Committee of the University of Sydney (2017/1284, 22 December 2017) and Sydney Local Hospital District Animal Welfare Committee (2014/007, 29 May 2014), in accordance with the Australian Code of Practice for the Care and Use of Animals for Scientific Purposes.

### 4.3. Cell Culture

The MO3.13 oligodendrocyte cell line was kindly provided by Prof Brett Garner, University of Wollongong, and cultured in DMEM medium supplemented with 10% fetal bovine serum and 2 mM L-glutamine. Cell culture reagents were purchased from Life Technologies. Serum-free oligodendrocyte differentiation medium was exactly as described by Emery and Dugas [58].

Cell viability was determined as the percentage of trypan blue-negative cells using a Countess Automated Cell Counter (Life Technologies, Mulgrave, VIC, Australia).

#### 4.3.1. Stable SphK2 Knock-Down

Short hairpin RNA (shRNA) sequences targeting SphK2 (A: CCGGCTACTTCTGCATCTACACCTACTCGAGTAGGTGTAGATGCAGAAGTAGTTTTTG, and B: CCGGCAGGATTGCGCTCGCTTTCATCTCGAGATGAAAGCGAGCGCAATCCTGTTTTTG) and lentiviral scramble control shRNA, in MISSION^®^ pLKO.1-puro Lentiviral vector (#SHC001), were purchased from Sigma Aldrich. The lentivirus was produced by co-transfection of HEK293T cells with pMD2.G (#12259), pMDLg/pRRE (#12251), and pRSV-Rev (#12253) viral packaging and envelope plasmids, supplied by Addgene (deposited by Dr. Didier Trono) [59]. Lentiviral supernatants were added to cultured MO3.13 cells for 72 h in the presence of 8 μg/mL polybrene (Sigma Aldrich, Castle Hill, NSW, Australia, #H9268). Stably-transfected cells were selected with 2.5 μg/mL puromycin (Life Technologies, Mulgrave, VIC, Australia, #A1113803) for 12 days before use as a cell pool.

#### 4.3.2. Metabolic Labelling with D7-Sphingosine

MO3.13 cells were grown to 80% confluency in 6-well plates, cultured overnight in serum-free OptiMEM I culture medium (#31985062, Life Technologies) supplemented with 2 mM L-glutamine, then incubated with 1 μM D7-sphingosine for 2 h. The medium was then replaced with fresh OptiMEM I to remove D7-sphingosine, and cells were incubated in quadruplicate for a further 0, 30, 60, or 120 min before washing once with PBS, then lysing in 250 μL of 20 mM Hepes, pH 7.4, 10 mM KCl, 1 mM dithiothreitol, 3 mM β-glycerophosphate, and cOmplete^TM^ EDTA-free protease inhibitor cocktail (Sigma Aldrich). Cells were lysed and homogenized by disruption in a Bioruptor Q800R2 Sonicator (QSonica, Newtown, CT, USA). Protein concentration was determined with the Pierce BCA assay (ThermoFisher Scientific). A total of 200 µL lysate protein was used for lipid extraction and sphingolipid quantification by targeted LC-MS/MS. Results were normalized for protein concentration. Experiments were performed on two stably transfected shSphK2 cell lines: shSphK2A and shSphK2B, compared to cells transfected with the control shRNA (shCtrl).

### 4.4. Lipid Extraction

Lipids were extracted from dissected hippocampus, liver tissue, and cell lysates using a two-phase procedure with methyl-tert-butyl ether (MTBE)/methanol/water (10:3:2.5, *v*/*v*/*v*) [60,61]. Hippocampus (10–20 mg) and liver tissue (5–10 mg) were pulverized over dry ice and transferred to 10 mL glass tubes containing 1.7 mL of MTBE, 500 µL of methanol, and 0.01% butylated hydroxytoluene, spiked with the internal standard mixture. Samples were sonicated in an ice-cold sonicating water bath (Unisonics, Sydney, Australia) for 3 × 30 min. Phase separation was induced by the addition of 417 µL of mass spectrometry-grade water, followed by vortexing and centrifugation at 1000× *g* for 10 min. The upper organic phase was collected in 5 mL glass tubes. The lower phase was re-extracted by adding 1 mL MTBE, 300 μL methanol, and 250 μL water. The organic phases were combined and dried under vacuum in a Savant SC210 SpeedVac (ThermoFisher Scientific), reconstituted in 300 μL methanol, and stored at −20 °C for subsequent analysis.

### 4.5. Sphingosine Kinase Activity Assays

Recombinant human SphK1 (5536-SK) and SphK2 (5298-SK) were purchased from R&D Systems, Minneapolis, MN, USA. Protein lysate from cortical tissue samples (20 mg) of SphK1^−/−^ and SphK2^−/−^ mice, along with WT littermates, was prepared as described previously (17). Reaction buffer was 50 mM Hepes, pH 7.4, 15 mM MgCl_2_, 10 mM KCl, 2 mM ATP, and 0.1% BSA. To each 25 µL reaction, either sphingosine, D7-sphingosine or sphinga-4,14-diene was added at increasing substrate concentrations (0.5, 1, 2, 5, 10, 20 µM). Reactions were started with the addition of recombinant SphK1 (10 pg/reaction) or SphK2 (20 pg/reaction) and run for 30 min at 37 °C. Reactions were stopped with 100 µL of methanol containing 100 pmoles of d17:1 sphingosine and d17:1 S1P as the internal standards for LC-MS/MS. Reaction products were quantified using LC-MS/MS, as described below.

### 4.6. Lipid Quantification by LC-MS/MS

#### 4.6.1. Untargeted Lipidomics

Lipid extracts from hippocampus tissue were analyzed on a Q-Exactive HF-X mass spectrometer with heated electrospray ionization (HESI) probe and Vanquish UHPLC system (ThermoFisher, Breman, Germany). Extracts were resolved on a 2.1 × 100 mm Waters Acquity C18 UPLC column (1.7 µm pore size), using a 25 min binary gradient at a 0.28 mL/min flow rate. HPLC gradient was as follows: 0 min, 70:30 A/B; 3 min, 70:30 A/B; 4.5 min, 57:43 A/B; 5.5 min, 45:55 A/B; 8 min, 35:65 A/B; 13 min, 15:85 A/B; 14 min, 0:100 A/B; 20 min, 0:100 A/B; 20.2 min, 70:30 A/B and 25 min, 70:30 A/B. Solvent A: 10 mM ammonium formate, 0.1% formic acid in acetonitrile:water (60:40); Solvent B: 10 mM ammonium formate, 0.1% formic acid in isopropanol:acetonitrile (90:10). Data were acquired in full scan/data-dependent MS2 (full scan resolution 70,000 FWHM, scan range 400–1200 *m*/*z*) in both positive and negative ionization modes. The ten most abundant ions in each cycle were subjected to fragmentation (MS2), with an isolation window of 1.4 *m*/*z*, collision energy 30 eV, resolution 17,500 FWHM, maximum integration time 110 ms and dynamic exclusion window 10 s. An exclusion list of background ions was used based on a solvent blank. An inclusion list of the [M+H]^+^ and [M-H]^−^ ions was used for all internal standards. LipidSearch software v4.1.30 (ThermoFisher) was used for lipid annotation, chromatogram alignment, and peak integration from extracted ion chromatograms. Lipid annotation was based on precursor (mass tolerance 4 ppm) and product ions in both positive and negative ion modes. Individual lipids were expressed as ratios to internal standard specific for each lipid class, then multiplied by the amount of internal standard added to produce a molar amount of each lipid per sample, which was normalized to milligrams extracted tissue.

#### 4.6.2. Targeted Lipidomics

Sphingolipid quantification was performed by selected reaction monitoring (SRM) on a TSQ Altis triple quadrupole mass spectrometer (ThermoFisher), operated in positive ion mode. Lipids were separated on a 2.1 × 100 mm Agilent Eclipse Plus C8 column (1.8 μm pore size) with a 24 min chromatography run at a flow rate of 300 µL/min. The HPLC gradient conditions were as follows: 0 min, 20:80 A/B; 2 min, 20:80 A/B; 7 min, 13:87 A/B; 14 min, 0:100 A/B; 20.5 min, 0:100 A/B; 21 min, 20:80 A/B; 24 min, 20:80 A/B. Solvent A: 0.2% formic acid, 2 mM ammonium formate in MilliQ water; Solvent B: 1% formic acid, 1 mM ammonium formate in methanol. Peak integration was carried out using Xcalibur software (ThermoFisher), and peaks were normalized as ratios to their class-specific internal standard (d18:1/12:0 SM, d18:1/12:0 HexCer, d18:1/12:0 sulfatide, d18:1/17:0 ceramide, d17:1 sphingosine, and d17:1 S1P). Specific SRM transitions used to identify each lipid are given in the relevant supplementary data tables. The *m*/*z* 264.3 and 262.3 product ions were used to distinguish lipids with a sphingosine or a sphingadiene base, respectively. In the case of SM species, both the dominant *m*/*z* 184.1 product ion and a second qualifying *m*/*z* 264.3 or 262.3 product ion were used for identification. All detected sphingolipids were verified as conforming to our previously identified quadratic elution profile [62]. For analysis of sphingosine, sphingadiene, S1P, and sphingadiene 1-phosphate, a previously-described method was used [23], with the inclusion of an *m*/*z* 298.3 → 262.3 transition for sphingadiene and *m*/*z* 378.3 → 262.3 for sphingadiene 1-phosphate.

For deuterated (D7-labelled) lipids, SRM lists were adjusted to account for additional mass of the deuterated sphingoid backbone (*m*/*z* + 7). Deuterated sphingosine and sphingadiene backbones were differentiated by the product ions *m*/*z* 271.3 and 269.3, respectively. SM species were verified by the presence of either *m*/*z* 271.3 or 269.3, along with the dominant *m*/*z* 184.1 product ion.

### 4.7. Lipid Characterization by 193 nm UVPD-MS/MS

One hundred and ninety-three-nanometer UVPD-MS/MS was implemented on a custom modified Q-Exactive Plus Orbitrap mass spectrometer (ThermoFisher), using the output from a Coherent ExciStar 500 XS ArF excimer laser (Santa Clara, CA, USA), as previously described [12]. Lipid solutions were prepared by placing 10 µL of lipid extracts into the wells of a PTFE 96-well plate. The samples were dried down in a GeneVac miVac sample concentrator (SP Scientific, Warminster, PA, USA) then resuspended in 40 µL propan-2-ol:methanol:chloroform (4:2:2, v:v:v) containing 2 mM lithium acetate. The plate was then sealed with Teflon Ultra-Thin Sealing Tape (Analytical Sales and Services, Pompton Plains, NJ, USA) before introduction to the mass spectrometer via nanoESI (nESI) using an Advion Triversa Nanomate (Ithaca, NY, USA) operating at a spray voltage of 1.3 kV and a gas pressure of 0.3 psi. The ion source interface was set to operate at an inlet temperature of 300 °C and S-Lens value of 50%. All nESI-UVPD-MS/MS spectra were acquired in the Orbitrap mass analyzer using a mass resolving power of 70,000 (at *m*/*z* 400). The automatic gain control (AGC) target was maintained at 1 × 10^5,^ and the maximum injection time was set to 500 ms. Precursor ions were mono-isotopically isolated using an isolation window of ± 0.5 *m*/*z*. UVPD-MS/MS spectra were generated using 100 laser pulses/scan (500 Hz repetition rate, approximately 4–5 mJ/pulse, max power 2.5 W), during which the higher-energy collisional dissociation (HCD) was set to 2 eV. Spectra shown are the average of 50 scans. The C=C double bond positions in sphingadiene-containing lipids were determined based on the diagnostic product ions formed from 193-nm UVPD fragmentation, as described previously [12].

### 4.8. Western Blotting

Cells were lysed in RIPA buffer (20 mM Tris-HCl, pH 7.4, 100 mM NaCl, 1 mM EDTA, 0.1% SDS, 0.5% sodium deoxycholate, 1% Triton X-100, 10% glycerol, 1 mM NaF, 2 mM Na4P2O7, cOmplete^TM^ EDTA-free protease inhibitor cocktail). RIPA buffer extracts (12.5 µg) were resolved on to 4–12% Bolt gels, then transferred to polyvinylidene fluoride membranes and blocked for 1 h with tris buffered saline containing 0.1% Tween-20 (TBST) and 5% skim milk powder. Membranes were washed 3 times with TBST, then cut in half at 50 kDa, and incubated overnight with rabbit-anti-SphK2 (Cell Signaling Technology, Beverly, MA, USA, #32346, 1:1000 dilution), rabbit anti-SphK1 (Cell Signaling Technology, #12071, 1:1000 dilution), or rabbit anti-actin (Abcam, #ab8227, 1:5000 dilution) in TBST with 5% bovine serum albumin. Membranes were washed 3 times with TBST, incubated for 1 h in TBST/5% skim milk with anti-rabbit-HRP (Cell Signaling Technology, #7074, 1:5000 dilution), washed another 3 times, then imaged using chemiluminescence reagent (Merck, Billerica, MA, USA, #WBKLS0500) and a BioRad ChemiDoc Touch imager. Densitometry was performed using BioRad ImageLab software version 5.2.

### 4.9. Statistical Analysis

For both untargeted and targeted lipidomic analysis, levels of each lipid were compared between SphK2^−/−^ and WT mice (or SphK1^−/−^ and WT) using a two-tailed unpaired *t*-test. Values were first log-transformed (natural log) to achieve a normal distribution for *t*-tests. The resultant *p*-values were adjusted for multiple comparisons using the false discovery rate approach of Benjamini, Krieger, and Yekutieli, with Q < 0.1 and 0.05 considered significant for untargeted and targeted LC-MS/MS, respectively (GraphPad PRISM). Complete lists of unadjusted *p*-values and adjusted Q-values are reported in Supplementary Tables S1–S4. For D7-sphingosine pulse–chase, the effect of incubation time and genotype (shCtrl vs. shSphK2) on the levels of each lipid were analyzed by 2-way ANOVA (GraphPad PRISM). Dunnett’s post-test was applied to test for differences at each time point relative to time = 0.

## Figures and Tables

**Table 1:** Lipid levels significantly different in the hippocampus of SphK2^−/−^ compared to wild-type mice.

Lipid	MW	Cal. *m*/*z*	Obs. *m*/*z*	Ion	WT Mean ± SD ^1^	SphK2^−/−^ Mean ± SD ^1^	*p* Value ^2^	Q Value ^3^
Cer(d18:2/18:0)	563.5277	564.5350	564.5343	H+	0.943 ± 0.410	4.558 ± 1.649	0.0001	0.0381
HexCer(d18:2/21:0-OH)	783.6224	784.6297	784.6297	H+	0.010 ± 0.006	0.064 ± 0.038	0.0003	0.0466
Cer(d18:2/22:0)	619.5903	620.5976	620.5953	H+	0.005 ± 0.002	0.016 ± 0.003	0.0004	0.0466
Cer(d18:2/22:1)	617.5747	618.5820	618.5818	H+	0.042 ± 0.028	0.152 ± 0.062	0.0011	0.0954
SM(d38:2)	756.6145	757.6218	757.6210	H+	0.049 ± 0.015	0.139 ± 0.060	0.0015	0.0954
SM(d36:2)	728.5832	729.5905	729.5903	H+	2.643 ± 1.259	7.277 ± 2.998	0.0016	0.0954
HexCer(d18:2/18:0)	725.5805	726.5878	726.5870	H+	0.028 ± 0.016	0.096 ± 0.050	0.0020	0.0954
Cer(d18:2/24:1)	645.6060	646.6133	646.6132	H+	0.008 ± 0.006	0.030 ± 0.013	0.0021	0.0954
HexCer(d18:2/18:0-OH)	741.5755	742.5828	742.5820	H+	0.031 ± 0.018	0.095 ± 0.057	0.0025	0.0954
HexCer(d18:2/20:0-OH)	769.6068	770.6141	770.6111	H+	0.045 ± 0.027	0.197 ± 0.129	0.0025	0.0954
Cer(d18:2/23:1)	631.5898	632.5976	632.5975	H+	0.018 ± 0.013	0.053 ± 0.019	0.0029	0.0954
Cer(d18:2/22:0-OH)	635.5853	636.5925	636.5922	H+	0.014 ± 0.010	0.042 ± 0.017	0.0030	0.0954
Cer(d18:2/24:2)	643.5903	644.5976	644.5974	H+	0.019 ± 0.012	0.052 ± 0.023	0.0034	0.0999

^1^ Mean and Standard Deviation (SD) have been reported in nmoles/mg tissue; ^2^ Unadjusted and ^3^ adjusted *p*-values for multiple comparisons (Q values) are reported. MW: Molecular weight; Cal. *m/z*: calculated mass-to-charge ratio; Obs. *m/z*: observed mass-to-charge ratio.

## Supplementary Materials

The following are available online at https://www.mdpi.com/2218-1989/10/6/236/s1, Table S1: Mean and standard deviation for 357 lipids identified in untargeted lipidomic analysis of hippocampus samples from SphK2^−/−^ mice and WT littermates at 8 months of age (six mice per group). Table S2: Mean and standard deviation for 363 lipids identified in untargeted lipidomic analysis of hippocampus samples from SphK1^−/−^ mice (n = 6) and WT littermates (n = 8) at 6 months of age. Results for Tables S1 and S2 are expressed as nmoles/mg tissue. Difference between sample means (T ratio), unadjusted (*p* value), and adjusted P values (Q values) are reported. Shaded lipid species are considered significant (Q < 0.1; Benjamini, Krieger, and Yekutieli false discovery rate approach). Table S3: Targeted analysis of sphingolipids in the hippocampus of a second cohort of SphK2^−/−^ mice (n = 9) and WT littermates (n = 11), at 6 months of age. Table S4: Targeted analysis of sphingolipids in the liver of SphK2^−/−^ mice (n = 6) and WT littermates (n = 6) at 8 months of age. Results for Tables S3 and S4 are expressed as pmoles/mg tissue ± SD. Unadjusted (*p* values) and adjusted (Q values) have been reported. Shaded lipid species are considered significant (Q < 0.05; Benjamini, Krieger, and Yekutieli false discovery rate approach).
